# Systemic complement activation in central serous chorioretinopathy

**DOI:** 10.1371/journal.pone.0180312

**Published:** 2017-07-03

**Authors:** Elon H. C. van Dijk, Roula Tsonaka, Ngaisah Klar-Mohamad, Diana Wouters, Aiko P. J. de Vries, Eiko K. de Jong, Cees van Kooten, Camiel J. F. Boon

**Affiliations:** 1 Department of Ophthalmology, Leiden University Medical Center, Leiden, the Netherlands; 2 Department of Medical Statistics and Bioinformatics, Leiden University Medical Center, Leiden, the Netherlands; 3 Department of Nephrology, Leiden University Medical Center, Leiden, the Netherlands; 4 Department of Immunopathology, Sanquin Research and Landsteiner Laboratory, Academic Medical Center, Amsterdam, the Netherlands; 5 Department of Medicine, Division of Nephrology and Transplantation, Leiden University Medical Center, Leiden, the Netherlands; 6 Department of Ophthalmology, Donders Institute for Brain, Cognition and Behaviour, Radboud University Medical Center, Nijmegen, the Netherlands; 7 Department of Ophthalmology, Academic Medical Center, University of Amsterdam, Amsterdam, the Netherlands; University of Manchester, UNITED KINGDOM

## Abstract

**Purpose:**

A clear link between several variants in genes involved in the complement system and chronic central serous chorioretinopathy (CSC) has been described. In age-related macular degeneration, a disease that shows clinical features that overlap with CSC, both genetic risk factors and systemic activation of the complement system have previously been found. In this case-control study, we assessed whether there is evidence of either systemic activation or inhibition of the complement system in patients with chronic CSC.

**Methods:**

A prospective case-control study of 76 typical chronic CSC patients and 29 controls without ophthalmological history was conducted. Complement activity assays (classical, alternative, and mannose-binding lectin pathway), complement factors 3, 4, 4A, 4B, B, D, H, I, and P, activation products C3d, C5a, and sC5b-C9, and the C3d/C3 ratio were analysed in either serum or plasma. A correction for possible effects of gender, age, body mass index, and smoking status was performed.

**Results:**

In this study, none of the tested variables, including regulation and activation products, proved to be significantly different between the groups. Moreover, no associations with either CSC disease activity or possible CSC related steroid use were observed.

**Conclusion:**

Despite the available literature regarding a possible relationship between chronic CSC and variants in genes involved in the complement system, we did not find evidence of an association of chronic CSC with either systemic complement activation or inhibition.

## Introduction

Central serous chorioretinopathy (CSC) mainly occurs in middle-aged male patients, and may cause irreversible vision loss.[1] CSC originates from dysfunction of the choroid, which shows an increase in permeability and thickness. These choroidal abnormalities and retinal pigment epithelium (RPE) damage lead to breakdown of the outer blood-retinal barrier, with subsequent serous subretinal fluid (SRF) leakage and neuroretinal detachment, often in the macula.[2–6] Although the use of exogenous corticoids is strongly associated with an increased risk for CSC,[7–10] the precise pathogenetic mechanism is unclear.[2–6]

The occurrence of CSC has been described in patients with several inflammatory diseases such as systemic lupus erythematosus and membranoproliferative glomerulonephritis.[11, 12] However, it is still unknown whether either one or both of these immune-mediated diseases or, alternatively, the prescribed glucocorticoid treatment leads to CSC in these patients.[13] Genetic factors may also play a part in the pathogenesis of CSC. Earlier reports on the familial occurrence of CSC have been published and associations between CSC and genetic variants in the *complement factor H* (*CFH*) gene, part of the innate immune system, have been found in several chronic CSC cohorts of diverse ethnic origins.[4, 14–18] Factor H, produced by both the choroidal and RPE cells, and critical in controlling local intraocular inflammation, is responsible for downregulation of the activation of the complement alternative pathway.[19–21] In the *CFH* gene, the single nucleotide polymorphisms (SNPs) have been observed to be either protective or risk conferring.[14–16] A recent study, demonstrating a possible involvement of the complement system in CSC, reported the absence of *complement component 4B* (*C4B*) gene copies to be associated with an increase in the risk of developing CSC, whereas the presence of 3 *C4B* copies is reported to be protective for CSC.[22]

In age-related macular degeneration (AMD), a disease with features overlapping with CSC,[15, 23] the Tyr402His amino acid substitution in the *CFH* gene has been shown to be strongly associated with the development of disease.[24] Moreover, a recently published study detected opposing effects of alleles in the *CFH* gene within a CSC and AMD patient group: genetic variants in *CFH* that led to an increased risk of AMD were protective for CSC, and vice versa.[15] In AMD, variants in *C3*, *CFB*, and *C2* have also been described to affect the risk of the disease and its progression.[25–27] Furthermore, in comparison with a control group systemic activation of the complement system has been detected in multiple AMD patient cohorts.[28, 29] Both in patients and in controls this activation showed a correlation with specific AMD risk alleles in complement genes, including the Tyr402His variant in *CFH*.[29]

Therefore, analogous to AMD, the association of complement gene SNPs in CSC may also point to a role for either increased or decreased systemic complement system activity in the pathogenesis of CSC. To assess systemic activation of the complement system in chronic CSC patients, we performed the first case-control study in this patient group and analysed whether there are differences in complement activation for several CSC subgroups.

## Materials and methods

### Study population

Seventy-eight chronic CSC patients who visited the Department of Ophthalmology at Leiden University Medical Center, the Netherlands, were included in this study. The study was powered based on previous reports on the C3d/C3 ratio as a measure of complement activation in AMD; with the current sample size we had >80% power to detect the previously observed effects.[28–30] Chronic CSC diagnosis was confirmed by fundoscopy, digital color fundus photography (Topcon Corp., Tokyo, Japan), fundus autofluorescence (Spectralis HRA+ optical coherence tomography (OCT); Heidelberg Engineering, Heidelberg, Germany), spectral-domain OCT (Spectralis HRA+OCT), fluorescein angiography (FA; Spectralis HRA+OCT), and indocyanine green angiography (Spectralis HRA+OCT), based on current knowledge from literature. All of the following characteristics had to be present: serous SRF on OCT, ≥1 area of multifocal diffuse leakage or irregular RPE window defects on FA, and corresponding hyperfluorescence on indocyanine green angiography. In all patients, either SRF or intraretinal edema on OCT had to have been present less than 2 years ago. Patients diagnosed with acute CSC as recognized by a focal leakage spot (ink blot) or a smokestack pattern on FA, patients with duration of disease of less than 3 months, and patients in whom either polypoidal choroidal vasculopathy or a choroidal neovascularisation or (signs of) AMD were present, were excluded.[2–5, 13, 28, 29, 31] Since the administration of corticosteroids can affect both the innate and adaptive immune system and can influence choroidal vascular permeability in male CSC patients by cadherin 5 downregulation, patients who used corticosteroids less than 1 year before diagnosis were also analysed separately in our study.[32, 33] Patients in whom the presence of SRF was confirmed on OCT at the day of blood puncture for this study, indicating active disease, were also analysed separately, to assess the possible influence of CSC disease activity on systemic complement activation. None of the patients had a history of either systemic autoimmune diseases associated with complement activation (systemic lupus erythematosus, ANCA-associated vasculitis, systemic sclerosis, rheumatoid arthritis) or with any (familial) ocular disease. Thirty-two matched controls without ophthalmological history were recruited at the outpatient clinic of the Department of Ophthalmology at Leiden University Medical Center.

Clinical data including demographics (age, gender, and ethnicity), body mass index (BMI), smoking, medical history, and use of both steroids and immunosuppressive medication were obtained, both for patients and controls (Table 1). For all patients, clinical information regarding CSC was collected. Written informed consent was obtained from all subjects before enrollment in this study. The study adhered to the tenets of the Declaration of Helsinki. Approval of the institutional review board and the ethics committee of Leiden University Medical Center were obtained (NL50816.058.14). Subjects were included in this study from June 2015 to April 2016.

**Table 1:** Demographic characteristics of the study population.

Variable	Patients (n = 76)	Controls (n = 29)	OR (95% CI)	P-value
Male gender (n [%])	70 (92%)	26 (90%)	1.78 (0.34–9.3)	0.50
Non-smoker (n [%])	35 (46%)	21 (73%)	reference	
Past smoker (n [%])	30 (39%)	2 (7%)	8.66 (1.85–40.6)	0.01
Current smoker (n [%])	11 (14%)	3 (10%)	2.47 (0.60–10.1)	0.21
Age (mean [SD], (in years))	49.2 (11.2)	43.0 (11.2)	1.06 (1.00–1.11)	0.045
Body mass index (mean [SD])	25.6 (3.30)	25.3 (4.36)	0.96 (0.84–1.10)	0.57

Only patients in whom all covariates were available were included in this study.

CI: confidence interval; OR: odds ratio; SD: standard deviation

### Complement measurements

After blood drawing, ethylenediaminetetraacetic acid (EDTA) samples were placed on ice and centrifuged (10 minutes at 1083 g at 4°C). These plasma samples were stocked in a -80°C freezer within 1 hour after collection. The activation products C5a and sC5b-C9 were measured in plasma samples using validated enzyme-linked immunosorbent assay kits (Hycult Biotech, Uden, the Netherlands).

After coagulation at room temperature for 1 hour, serum samples were centrifuged (10 minutes at 1083 g at 4°C) and aliquots were immediately placed in the -80°C freezer. These serum samples were used to quantify the complement activity of the classical pathway (CP50), the alternative pathway (AP50), and the mannose-binding lectin pathway (LP50) with the Wieslab kit (Euro-diagnostics, Malmö, Sweden). In addition, complement factors 3, 4, B, D, H, I, P, the activation product C3d, and the C3d/C3 ratio (parameter of activation of complement alternative pathway factor 3) were analysed in these sera, according to previously described measurement techniques.[29] Moreover, complement factors 4A and 4B were assessed separately, according to a protocol that has been previously published.[34]

### Statistical analysis

Statistical analyses were performed in R (R Foundation for Statistical Computing, Vienna, Austria). Since the information for the covariates age, gender, BMI, and smoking was not complete for 5 subjects, 105 subjects (76 patients, 29 controls) could be included in the statistical tests. Baseline and clinical characteristics, and values of systemic complement activation of both cases and controls were described by using standard descriptive statistics. Mean differences between the case and control group were assessed using a linear regression model, where correction for the covariates was performed. A role for possible CSC related steroid use and CSC disease activity was also assessed by using a linear regression model, for which again was corrected for the previously mentioned covariates.

Two-sided p-values of <0.05 were considered to be statistically significant. The Bonferroni correction was performed for the tests comparing different patient groups and control subjects, since adjusting was required for the 16 tests that were done.

## Results

### Patient characteristics

Since information regarding all covariates was available for 76 patients, only the assessments of these chronic CSC patients could be taken into account. The mean age of these patients (70 males, 6 females) was 49 ± 11 years (range, 25–83 years). Sixty patients (79%) were Caucasian. Either bilateral SRF on OCT or bilateral ‘hot spots’ of leakage on FA either was or had been present in 28 patients (37%), and until blood puncture for this study a recurrence of CSC had been diagnosed in 33 patients (43%). Medical history of 12 patients (16%) revealed hypertension, and in 3 other patients (4%) other cardiovascular diseases had been previously diagnosed. Three patients (5%) reported that they were previously clinically diagnosed with a depression. In 3 patients (5%) a burn-out had been diagnosed, whether in 2 other patients (3%) this was the case for post-traumatic stress disorder. None of the included patients reported the use of immunosuppressive medication. Fifteen chronic CSC patients (20%) had used corticosteroids less than 1 year before diagnosis, and in 23 patients (30%) the presence of SRF on OCT was confirmed at the day of blood puncture for this study, indicating active disease.

### Control characteristics

The mean age of the 29 control subjects (26 males, 3 females; 83% of Caucasian ethnicity), in whom no ophthalmological diseases had been diagnosed before and from whom covariates were available, was 43 ± 11 years (range, 24–52 years), which was significantly lower compared to the patient group (p = 0.04). When comparing the patients and controls, no differences regarding gender, BMI, and current smoking could be detected. Three controls (10%) were previously diagnosed with hypertension, and 2 others (7%) were known with other cardiovascular diseases. Two controls (7%) reported that a depression had been diagnosed in their medical history, whereas 3 others (10%) reported the diagnosis of a burn-out. Four controls (14%) reported the previous use of steroids.

### Complement levels

After the Bonferroni correction had been performed and covariates had been taken into account (Table 1), no significant differences were detected between CSC patients and the control group for the classical, alternative, and mannose-binding lectin pathway. Moreover, no significant differences were found for complement factors 3, 4, 4A, 4B, B, D, H, I, and P, activation products C3d, C5a, and sC5b-C9, and the C3d/C3 ratio (Table 2). To detect possible pathophysiological differences between patient groups, dividing CSC patients in groups based on a possible relationship with either steroid use (Table 3) or CSC disease activity at the day of blood puncture (Table 4), also did not lead to differences in either activation or inhibition of the complement system comparing patients and controls.

**Table 2:** Mean complement activities and concentrations in chronic central serous chorioretinopathy (CSC) patients and controls.

Complement activity/protein (units)	Chronic CSC (n = 76), mean (SD)	Controls (n = 29), mean (SD)	P-value	Adjusted p-value	Normal laboratory values
Classical pathway activity (CP50) (%)*	101.2 (4.23)	102.2 (2.85)	0.05	0.81	>74
Alternative pathway activity (AP50) (%)*	89.8 (18.6)	90.6 (11.7)	0.15	1.00	>39
Mannose-binding lectin pathway (LP50) (%)*	68.7 (41.0)	65.1 (44.4)	0.95	1.00	>10
C3 (mg %)	126.6 (24.4)	122.6 (20.2)	0.95	1.00	90–200
C4 (mg %)	26.3 (8.06)	24.2 (7.06)	0.13	1.00	9.5–41.5
C4A (μg/ml)	299.7 (165.7)	335.7 (151.9)	0.17	1.00	NA
C4B (μg/ml)	115.2 (34.6)	97.3 (26.6)	0.05	0.82	NA
CFB (mg %)	17.3 (3.74)	16.4 (2.89)	0.41	1.00	13–22
CFD (μg/ml)	2.75 (0.62)	2.68 (0.68)	0.78	1.00	NA
CFH (mg %)	21.6 (3.43)	21.0 (3.54)	0.79	1.00	19–26
CFI (mg %)	45.9 (7.78)	46.0 (7.39)	0.64	1.00	NA
CFP (μg/ml)	23.1 (6.45)	24.1 (4.25)	0.73	1.00	17.1–27.7
C3d (μg/ml)	2.61 (0.97)	2.76 (1.49)	0.56	1.00	NA
C5a (ng/ml)	5.32 (12.6)	3.16 (3.35)	0.22	1.00	NA
C5b-C9 (Au/ml)	0.70 (0.18)	0.70 (0.18)	0.47	1.00	NA
C3d/C3 ratio	0.21 (0.09)	0.23 (0.13)	0.73	1.00	NA

Only patients in whom all covariates were available were included in this study.

* Determined by ELISA, and presented as a percentage of the standard in the kit.

ELISA: enzyme-linked immunosorbent assay; NA: not available; SD: standard deviation

**Table 3:** Mean complement activities and concentrations in chronic central serous chorioretinopathy (CSC) patients and controls. Chronic CSC patients were divided into 2 groups: patients who had used steroids within 1 year before diagnosis versus patients who had not used steroids within 1 year before diagnosis.

Complement activity/protein (units)	Chronic CSC, steroid related (n = 15), mean (SD)	Chronic CSC, non-steroid related (n = 61), mean (SD)	Controls (n = 29), mean (SD)	P-value	Adjusted p-value
Classical pathway activity (CP50) (%)*	102.4 (3.85)	101.0 (4.30)	102.2 (2.85)	0.08	1.00
Alternative pathway activity (AP50) (%)*	87.9 (21.3)	90.2 (18.0)	90.6 (11.7)	0.29	1.00
Mannose-binding lectin pathway (LP50) (%)*	68.1 (46.0)	68.8 (40.1)	65.1 (44.4)	0.99	1.00
C3 (mg %)	126.8 (25.8)	126.5 (24.3)	122.6 (20.2)	1.00	1.00
C4 (mg %)	29.0 (8.55)	25.7 (7.87)	24.2 (7.06)	0.15	1.00
C4A (μg/ml)	303.6 (131.4)	298.7 (174.0)	335.7 (151.9)	0.38	1.00
C4B (μg/ml)	116.6 (41.0)	114.9 (33.2)	97.3 (26.6)	0.14	1.00
CFB (mg %)	16.9 (4.55)	17.3 (3.55)	16.4 (2.89)	0.63	1.00
CFD (μg/ml)	2.73 (0.44)	2.75 (0.65)	2.68 (0.68)	0.92	1.00
CFH (mg %)	22.3 (4.46)	21.5 (3.14)	21.0 (3.54)	0.80	1.00
CFI (mg %)	43.0 (9.24)	46.6 (7.30)	46.0 (7.39)	0.42	1.00
CFP (μg/ml)	21.7 (6.84)	23.5 (6.36)	24.1 (4.25)	0.61	1.00
C3d (μg/ml)	2.40 (0.66)	2.66 (1.03)	2.76 (1.49)	0.34	1.00
C5a (ng/ml)	2.09 (0.75)	6.11 (14.0)	3.16 (3.35)	0.01	0.22
C5b-C9 (Au/ml)	0.64 (0.15)	0.71 (0.18)	0.70 (0.18)	0.15	1.00
C3d/C3 ratio	0.20 (0.08)	0.22 (0.09)	0.23 (0.13)	0.75	1.00

Only patients in whom all covariates were available were included in this study.

* Determined by ELISA, and presented as a percentage of the standard in the kit.

ELISA: enzyme-linked immunosorbent assay; SD: standard deviation

**Table 4:** Mean complement activities and concentrations in chronic central serous chorioretinopathy (CSC) patients with subretinal fluid at the day of blood taking, compared to controls.

Complement activity/protein (units)	Active chronic CSC (n = 22), mean (SD)	Controls (n = 29), mean (SD)	P-value	Adjusted p-value
Classical pathway activity (CP50) (%)*	101.2 (3.10)	102.2 (2.85)	0.19	1.00
Alternative pathway activity (AP50) (%)*	85.2 (19.3)	90.6 (11.7)	0.12	1.00
Mannose-binding lectin pathway (LP50) (%)*	71.5 (34.3)	65.1 (44.4)	0.43	1.00
C3 (mg %)	127.4 (26.2)	122.6 (20.2)	0.51	1.00
C4 (mg %)	27.4 (8.86)	24.2 (7.06)	0.07	1.00
C4A (μg/ml)	252.1 (135.5)	335.7 (151.9)	0.04	0.62
C4B (μg/ml)	107.7 (36.5)	97.3 (26.6)	0.31	1.00
CFB (mg %)	17.2 (4.21)	16.4 (2.89)	0.57	1.00
CFD (μg/ml)	2.61 (0.51)	2.68 (0.68)	0.89	1.00
CFH (mg %)	21.3 (3.43)	21.0 (3.54)	1.00	1.00
CFI (mg %)	44.8 (9.51)	46.0 (7.39)	0.95	1.00
CFP (μg/ml)	23.39 (6.67)	24.1 (4.25)	0.76	1.00
C3d (μg/ml)	2.64 (0.95)	2.76 (1.49)	0.83	1.00
C5a (ng/ml)	5.72 (16.6)	3.16 (3.35)	0.43	1.00
C5b-C9 (Au/ml)	0.68 (0.22)	0.70 (0.18)	0.57	1.00
C3d/C3 ratio	0.21 (0.09)	0.23 (0.13)	0.84	1.00

Only patients in whom all covariates were available were included in this study.

* Determined by ELISA, and presented as a percentage of the standard in the kit.

ELISA: enzyme-linked immunosorbent assay; SD: standard deviation

## Discussion

To the best of our knowledge, we conducted the first case-control study on systemic complement activation in chronic CSC patients. Although the study was sufficiently powered to detect differences in the C3d/C3 ratio as previously reported in AMD,[28, 29] no association was found between CSC and both complement activation and inhibition, which suggests that the effect size of C3d/C3 in CSC is either smaller, or absent. Moreover, when dividing the patients into subgroups based on either possible CSC related steroid use or CSC disease activity, no differences were detected either between several patient groups or between patients and controls for the sample sizes that were included in this study.

The outcome of our study differs from findings in AMD, a disease that shows overlapping features with CSC and in which systemic activation of the complement system has been found.[15, 23, 28, 29] In a recent study assessing SNPs in complement genes in both AMD and CSC patients, opposing effects were observed for genetic associations of the *CFH* gene, suggesting that the complement system is involved in CSC, although the direction of the effect remained uncertain.[15] The lack of any association in our study may be a consequence of the fact that the effect sizes for genetic associations of *CFH* in CSC were weaker compared to AMD.[15, 28, 29] Variables known to influence activation of the complement system, such as age, gender, BMI, and smoking, were taken into account during statistical analysis of data from our study.[28, 29]

Since steroid use has been described to be the most pronounced risk factor for CSC, and the etiology in patients with previously reported CSC related steroid has been described to differ from non-steroid associated disease, both groups were also analysed separately.[9, 33] Our results indicate that there is no clear role for complement activation in either of these patient groups. From all patients, in whom SRF had to have been present on OCT within the last 2 years, patients with active disease at the day of blood puncture were also analysed separately. Even in cases with active CSC at the time of systemic complement analysis, no abnormalities in complement factors were detected in this study, which would indicate that systemic complement dysfunction does not play a significant role in active CSC as well.

Despite the available literature regarding a possible relationship between chronic CSC and variants in genes involved in the complement system, an association between chronic CSC and either systemic complement activation or inhibition was not found in this study. However, for several complement components the number of included patients and controls could have led to underpowered results. In future studies, the exact role of the complement system in CSC remains to be elucidated. Since previously reported genetic associations clearly suggest involvement of the complement system in CSC, the functional translation of these findings and their contribution to the disease mechanism should be the focus of future investigations.

## Supporting information

S1 File Raw complement activity and concentration data, and information on confounders in chronic central serous chorioretinopathy patients and controls. (XLSX)
